# Maternal Protein Restriction Inhibits Insulin Signaling and Insulin Resistance in the Skeletal Muscle of Young Adult Rats

**DOI:** 10.14789/jmj.JMJ23-0029-OA

**Published:** 2024-03-18

**Authors:** KENTARO AWATA, HIROMICHI SHOJI, YOSHITERU ARAI, IRENA SANTOSA, KAZUHIDE TOKITA, YAYOI MURANO, TOSHIAKI SHIMIZU

**Affiliations:** 1 Department of Pediatrics and Adolescent Medicine, Juntendo University Graduate School of Medicine, Tokyo, Japan; 1 Department of Pediatrics and Adolescent Medicine, Juntendo University Graduate School of Medicine, Tokyo, Japan; 2 Department of Pediatrics Medicine, Juntendo University Faculty of Medicine, Tokyo, Japan; 2 Department of Pediatrics Medicine, Juntendo University Faculty of Medicine, Tokyo, Japan

**Keywords:** fetal growth restriction, insulin resistance, skeletal muscle, insulin signaling, DNA methylation

## Abstract

**Objectives:**

Infants with fetal growth restriction (FGR) are at a risk of developing metabolic syndromes in adulthood. We hypothesized that skeletal muscle degeneration by nutrition-restricted FGR results in abnormal insulin signaling and epigenetic changes.

**Material and Methods:**

To develop a protein-restricted FGR model, rats were fed a low-protein diet (7% protein) during the gestational period; rats fed a normal diet (20% protein) were used as controls. At 8 and 12 weeks of age, the pups were subjected to oral glucose tolerance test (OGTT) and insulin tolerance test (ITT) to evaluate insulin resistance. At 12 weeks, the mRNA and protein levels of insulin signaling pathway molecules in the skeletal muscles were examined. DNA methylation of promoters was detected. DNA extracted from skeletal muscles was used as a template for methylation-specific PCR analysis of *GLUT4*.

**Results:**

The body weight of FGR rats from birth to 8 weeks was significantly lower than that of the controls; no significant difference was observed between the groups at 12 weeks. In the OGTT and ITT, the incremental area under the curve (iAUC) was significantly higher in FGR rats than in the controls at 12 weeks. The mRNA and protein levels of Akt2 and GLUT4 in the plantar muscles were significantly lower in FGR rats than in the controls. *GLUT4* methylation was comparable between the groups.

**Conclusions:**

Protein-restricted FGR rats showed insulin resistance and altered insulin signaling in skeletal muscles after 12 weeks. However, we could not demonstrate the involvement of DNA methylation in this model.

## Introduction

Fetal growth restriction (FGR) is known to increase predisposition to a variety of chronic diseases such as hypertension, cardiovascular diseases, obesity, insulin resistance/type 2 diabetes mellitus (T2DM), and other metabolic syndromes in adulthood^1, 2)^. Fetal growth is dependent on the continuous transfer of nutrients and oxygen from the mother via the placenta. Maternal undernutrition represents a global problem as it is associated with the incidence of chronic diseases in newborns; it also affects the development of newborns^3)^. Barker and Hales first proposed the “thrifty phenotype” hypothesis, according to which fetal undernutrition is strongly associated with numerous chronic conditions later in life^4)^.

The skeletal muscle is the primary site of insulin-stimulated glucose uptake, accounting for up to 70% of whole-body glucose disposal^5)^, and is a key regulator of whole-body energy metabolism^6)^. Furthermore, the skeletal muscle is among the tissues that are most sensitive to maternal nutritional restriction^7)^. Upon binding to its receptor, insulin facilitates glucose uptake in skeletal muscle mainly through glucose transporters such as glucose transporter isoform 4 (GLUT4). In this process, distinct signaling cascades that include multiple enzymes, such as the phosphoinositide 3-kinase (PI3K)/protein kinase B (also known as Akt) pathway, are involved^8, 9)^. PI3K binds to insulin receptor substrate (IRS) proteins, resulting in the phosphorylation and activation of Akt, which translocate GLUT4 to the plasma membrane, enabling glucose uptake into the skeletal muscle.

Several FGR rat models have been established to investigate the mechanisms underlying intrauterine events and the eventual adult phenotype^10-13)^. The maternal protein-restricted FGR model is one of the most extensively studied models, and the outcomes of offspring bear striking similarities to human diabetes, at both whole body and molecular levels^14)^. Recently, it has been proposed that the epigenetic regulation of genes, particularly the methylation of clusters of CpG dinucleotides (islands) in the promoter regions of certain genes, may contribute to metabolic reprogramming^15)^. Lillycrop et al.^16)^ demonstrated that feeding a protein-restricted diet to pregnant rats increased glucocorticoid receptor and peroxisome proliferator-activated receptor α (PPARα) expression in the livers of offspring by inducing the hypomethylation of constitutive promoters. These findings suggest that an epigenetic mechanism induced by prenatal nutrition may generate an altered phenotype in the offspring^17)^.

We hypothesized that the degeneration of skeletal muscle by FGR results in epigenetic changes and abnormal insulin signaling, which leads to the development of diabetes mellitus without obesity. The study was performed using a rat model of maternal protein-restricted FGR.

## Materials and Methods

### Animals and experimental designs

Female Sprague-Dawley rats (gestational day 11) were purchased from Sankyo Labo Service Corporation, Inc. (Tokyo, Japan) and housed in individual cages in the same room at 24-25°C and 60% relative humidity under a 12-:12-h light-dark cycle with free access to food and water at Juntendo University Animal Care Facility (Tokyo, Japan). Pregnant rats were fed either a diet containing 20% protein (control group) or an isocaloric diet containing 7% protein (FGR group) until delivery. After delivery, each maternal rat was fed a normal diet during the 21-d lactation period. At 21 d of age, all offspring were fed a normal diet. In this study, only the male offspring were used to avoid the effects of sex and hormone differences. The study protocol was approved by the Animal Care Committee of Juntendo University (1455).

The control and FGR groups comprised six offspring each. We measured the body weight of the offspring at birth and at 4, 8, and 12 weeks of age. The oral glucose tolerance test (OGTT) and insulin tolerance test (ITT) were performed at 8 and 12 weeks of age, and dissection was performed at 12 weeks of age. Anesthesia was induced with 2%-2.5% isoflurane to reduce pain before dissection. The rat aorta was punctured and the organs were thoroughly perfused with saline to remove red blood cells. Thereafter, the soleus, gastrocnemius, and plantar muscles of the lower limbs were harvested. These three skeletal muscles were immersed in RNAlater liquid (Gene Keeper RNA & DNA stabilization solution; Nippon gene Co., Ltd, Tokyo, Japan) or snapped in liquid nitrogen and stored at -80 °C until further analysis.

### Oral glucose tolerance test

Body weight and blood glucose and insulin levels were measured in overnight-fasted rats. After the initial blood collection, glucose solution (2 g/kg) was administered via oral gavage. Blood glucose level was measured at 15, 30, 60, 90, and 120 min after glucose administration using Precision Xceed (cat. no. 71085-80; ABBOTT Japan, Chiba, Japan). Blood insulin level was measured at 30, 60, and 120 min using an Ultra-sensitive Rat Insulin ELISA kit (cat. no. 49170-51; Morinaga Institute of Biological Science, Inc., Kanagawa, Japan). Blood samples for the analyses were collected from the tail veins, and the procedures were performed without sedation. Incremental areas under the curve (iAUCs) for both plasma insulin and glucose levels were calculated.

### Insulin tolerance test

Body weight and blood glucose and insulin levels were measured in overnight-fasted rats. After the initial blood collection, insulin (0.5 IU/kg) was administered via intraperitoneal injection, and blood samples were collected at 0, 30, 60, and 120 min to measure plasma glucose level. The iAUC for the plasma glucose level was then calculated.

### Real-time quantitative reverse transcription- polymerase chain reaction

Real-time quantitative reverse transcription-polymerase chain reaction (RT-qPCR) was performed to assess the expression of insulin signaling pathway molecules (*Akt2*, *PI3K*, *IRS1*, and *GLUT4*) in the skeletal muscles (plantar, soleus, and gastrocnemius) using the TaqMan^®^ system (Applied Biosystems, Woburn, MA, USA) according to the manufacturer's instructions. The skeletal muscles were crushed, and RNA was extracted using the RNeasy^®^ Mini Kit (cat. no. 74104; QIAGEN N.V., Hilden, Germany). The mRNA expression levels of *PI3K*, *Akt2*, *GLUT4*, and *IRS1* were normalized to those of the housekeeping gene *β-actin*. The relative expression levels of target genes were calculated using the 2^-^^ΔΔCq^ method. Primers and probes for *SiC2a4* (*GLUT4*) (Rn01752377_m1; Applied Biosystems, Foster City, CA, USA), *Akt2* (Rn00690901_m1), *PiK3cg* (Rn00667869_m1), *IRS1* (Rn02132493_s1), and *β-actin* (Rn00667869_m1) were prepared using TaqMan gene expression assays.

### Western blotting

The frozen skeletal muscle tissues were crushed using a homogenizer (TissueLyser II; Qiagen, Hilden, NRW Germany). Proteins were extracted from the precipitate using radioimmunoprecipitation assay buffer (50 mmol/L Tris-HCl buffer (pH 7.6), 150 mmol/L NaCl, 1% Nonidet^®^ P40, 0.5% sodium deoxycholate, protease inhibitor cocktail, and 0.1% SDS) (cat. no. 08714-04; Nacalai Tesque, Kyoto, Japan), supplemented with phosphatase inhibitor cocktail (cat. no. 07574-61; Nacalai Tesque). Protein concentrations were quantified using the Pierce™ BCA Protein Assay Kit (cat. no. 23225; Thermo Fisher Scientific, Waltham, MA, USA). Polyvinylidene fluoride (PVDF) membrane was blocked with Bullet Blocking One for western blotting (cat. no. 13779-56; Nacalai Tesque) for 5 min, and then incubated overnight at 5°C with the following primary antibodies: rabbit anti-Akt2 monoclonal antibody (1:1000; cat. no. 9272s; Cell Signaling Technology, Danvers, MA, USA), mouse anti GLUT4 monoclonal antibody (1:1000; cat. no. 2213; Cell Signaling Technology), and rabbit anti-GAPDH monoclonal antibody (1:10000; cat. no. 5174S; Cell Signaling Technology). GAPDH was used as the internal reference. The PVDF membrane was washed three times with Tris-buffered saline containing 0.05% Tween-20 (TBST) and incubated with horseradish peroxidase (HRP)-conjugated goat anti-rabbit IgG (1:10000; cat. no. 7074; Cell Signaling Technology) or HRP-conjugated goat anti-mouse IgG (1:10000; cat. no. 7076; Cell Signaling Technology) for 1 h at approximately 25°C. Subsequently, the PVDF membrane was washed three times with Tris Buffered Saline with Tween 20 (TBST), and the blots were developed using ImmunoStar LD (cat. no. 296-69901; FUJIFILM Wako Pure Chemical Corporation, Osaka, Japan) and the intensity of the bands was quantified using FUSION software (Vilber Lourmat, Collegien, France).

### DNA methylation detection

DNA methylation in the promoters was detected using bisulfate sequencing PCR. Genomic DNA extracted from rat skeletal muscle was used as a template for methylation-specific PCR analysis of the target gene (*GLUT4*). All primers were designed according to previous studies^17, 18)^.

### Statistical analysis

Results are presented as mean ± standard deviation. Differences between the groups were compared using Mann-Whitney *U* test. Pearson’s correlation analysis was used to analyze the association between insulin and protein levels and FGR. Statistical significance was set at *p* < 0.05. Kendall rank correlation coefficient was used to analyze the association between insulin signaling and iAUC. All statistical analyses were performed using GraphPad Prism V.7.02 (GraphPad Software, San Diego, CA, USA).

## Results

### Weight trajectories of the FGR and control rats

**Figure 1:**
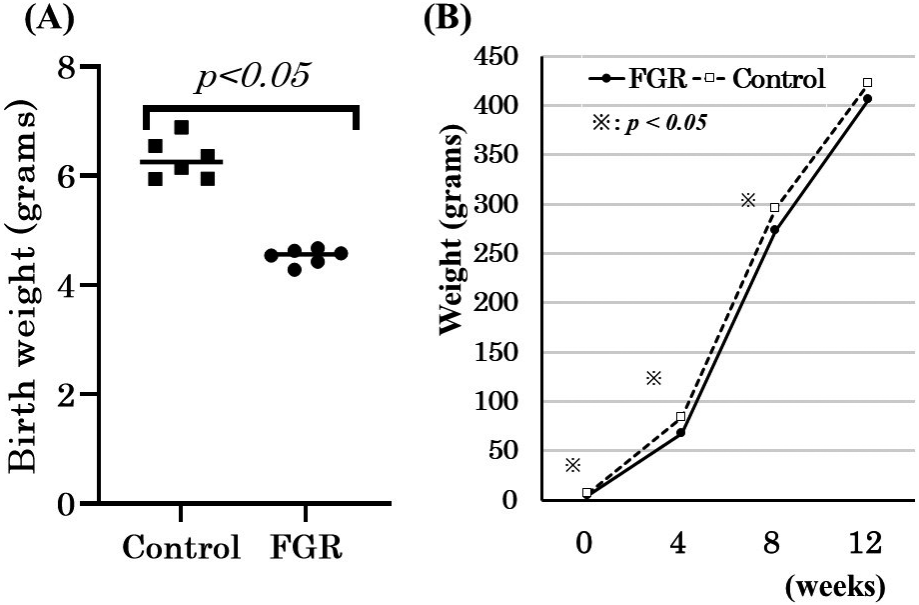
Weights of fetal growth restriction (FGR) and control rats from birth (A) to 12 weeks of age (B). Data are shown as mean ± SD. ※ *p* < 0.05 vs. control rats.

The mean birthweight of rats in the FGR group (4.4 ± 0.4 g, n = 6) was lower than that of rats in control group (6.3 ± 0.7 g, n = 6) (*p* < 0.05). The mean body weight of rats in the FGR group was also significantly lower than that of rats in the control group until 8 weeks of age. However, no significant difference was observed between the groups at 12 weeks of age (Figure 1).

### Oral glucose tolerance test results

**Figure 2:**
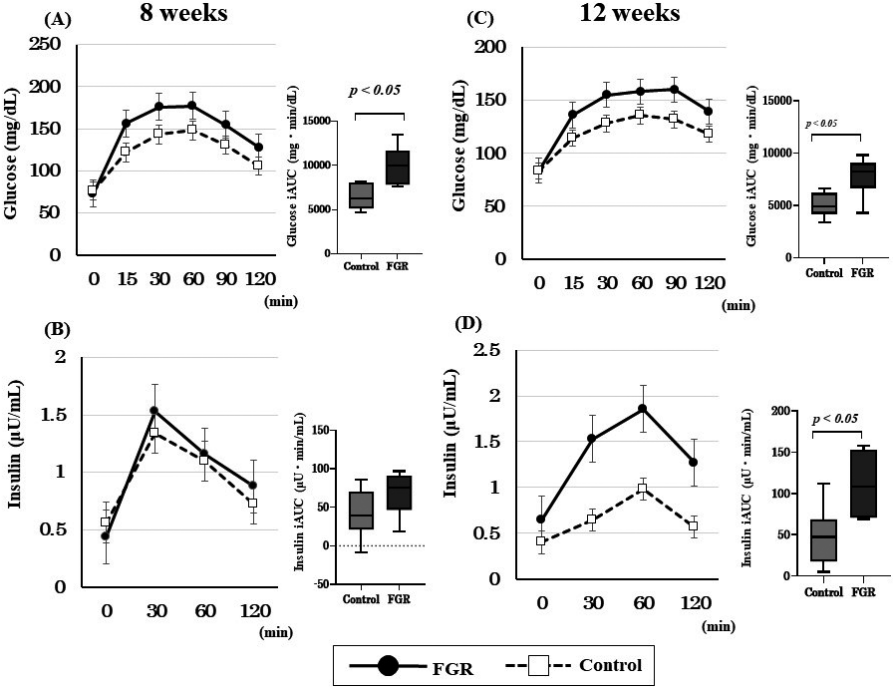
Results of oral glucose tolerance test (OGTT) at 8 and 12 weeks of age (A) Glucose level and glucose incremental area under the curve (iAUC) at 8 weeks. (B) Insulin level and insulin iAUC at 8 weeks. (C) Glucose level and glucose iAUC at 12 weeks. (D) Insulin level and insulin iAUC at 12 weeks.

At 8 weeks of age, the iAUC 0-120 min of the blood glucose level of the FGR group was 9996 ± 3451 mg min^-^^1^ dL^-^^1^, which was higher than that of the control group (6448 ± 1768 mg min^-^^1^ dL^-^^1^) (*p* < 0.05). However, the iAUC of insulin level was not significantly different between the groups (68.51 ± 49.85 mg min^-^^1^ dL^-^^1^ for the FGR group and 42.14 ± 43.42 mg min^-^^1^ dL^-^^1^ for the control group; *p* = 0.166) (Figure 2). The iAUC 0-120 min of the blood glucose level at 12 weeks of the FGR group was 7786 ± 3511 mg min^-^^1^ dL^-^^1^, which was higher than that of the control group (5034 ± 1689 mg min^-^^1^ dL^-^^1^) (*p* < 0.05). Similarly, the iAUC of insulin level was significantly different between the groups (110.9 ± 47.1 mg min^-^^1^ dL^-^^1^ in the FGR group and 47.93 ± 69.89 mg min^-^^1^ dL^-^^1^ in the control group; *p* < 0.05) (Figure 2).

### Insulin tolerance test results

**Figure 3:**
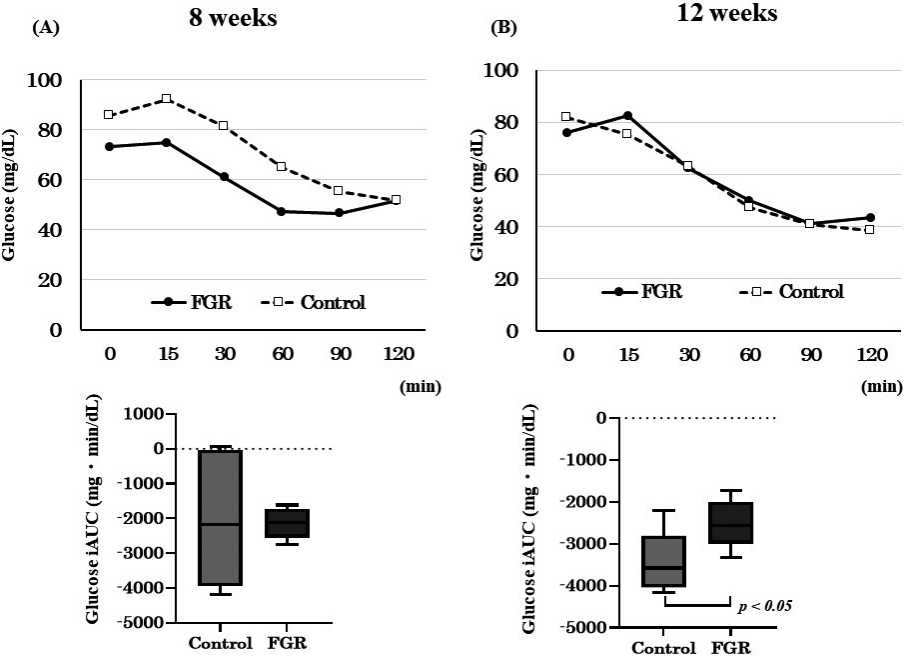
Results of insulin tolerance test (ITT) at 8 and 12 weeks of age (A) Glucose level and glucose incremental area under the curve (iAUC) at 8 weeks. (B) Glucose level and glucose iAUC at 12 weeks.

At 8 weeks of age, the iAUC 0-120 min of the blood glucose level was -2146 ± 599 (mg min^-^^1^ dL^-^^1^) for the FGR group and -2070 ± 2108 (mg min^-^^1^ dL^-^^1^) for the control group; there was no significant difference between the groups. The iAUC 0-120 min of the blood glucose level of the FGR group at 12 weeks of age (-2530 ± 807 mg min^-^^1^ dL^-^^1^) was significantly lower than that of the control group (-3421 ± 1216 mg min^-^^1^ dL^-^^1^; *p* < 0.05) (Figure 3).

### Insulin signaling in the skeletal muscles

**Figure 4:**
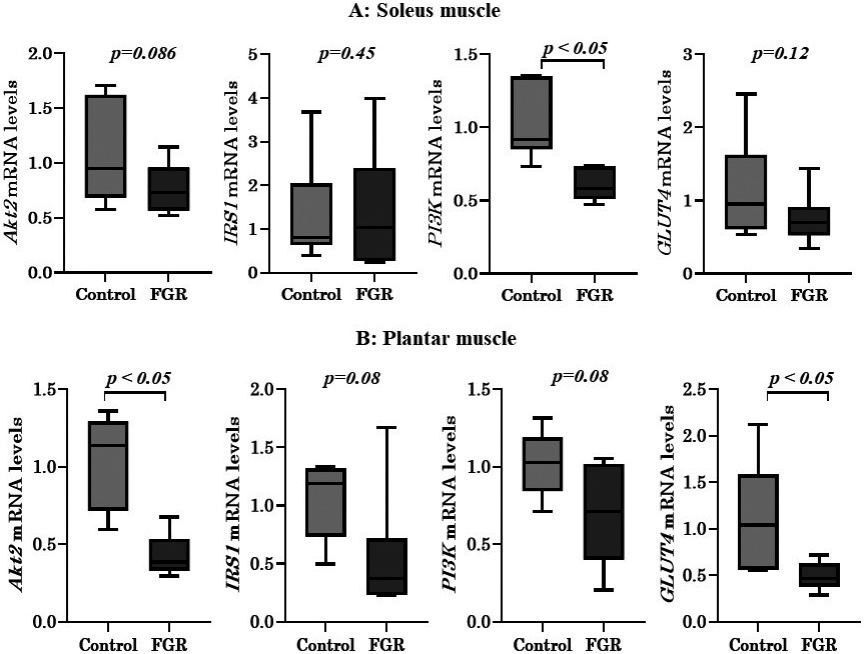
Results of real-time polymerase chain reaction analysis of protein kinase B (*Akt2*), phosphoinositide 3-kinases (*PIK3*), insulin receptor substrate 1 (*IRS1*), and glucose transporter type 4 (*GLUT4*) mRNA in the (A) soleus muscle and (B) plantar muscle of fetal growth restriction (FGR) and control rats. ※ *p* < 0.05 vs. control rats.

The mRNA expression of *PI3K* in the soleus muscle and that of *Akt2* and *GLUT4* in the plantar muscles were lower in the FGR group (*p* < 0.05) than in the control. There were no significant differences in the expression levels of genes encoding other insulin signaling pathway molecules (Figure 4).

### Western blot analysis of insulin signaling molecules in the skeletal muscles

**Figure 5:**
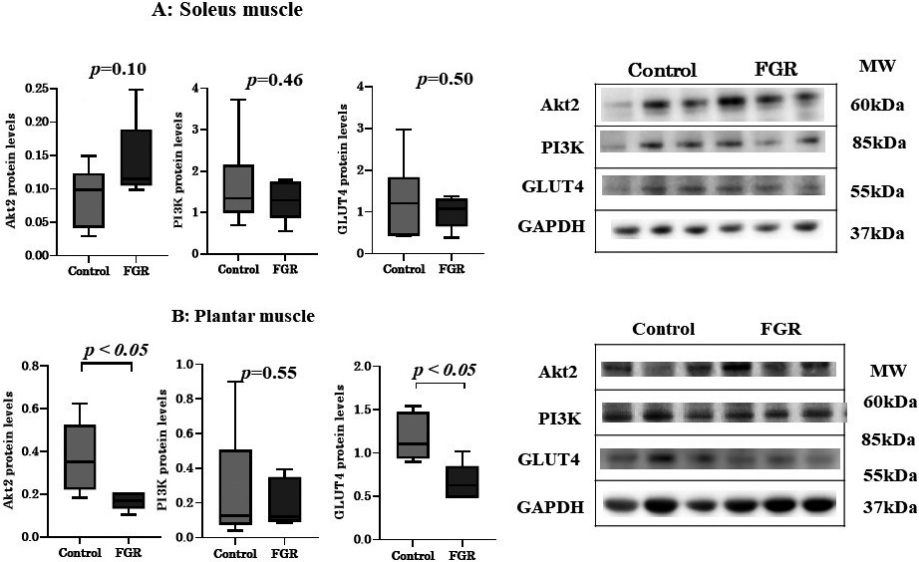
Results of western blot analysis of protein kinase B (Akt2), phosphoinositide 3-kinases (PIK3), and glucose transporter type 4 (GLUT4) in the (A) soleus muscle and (B) plantar muscle of fetal growth restriction (FGR) rats and control rats. ※ *p* < 0.05 vs. control rats.

The protein levels of Akt2 and GLUT4 in the plantar muscles were lower in the FGR group (*p* < 0.05) than in the control. There were no significant differences in the protein levels of other insulin signaling pathway molecules (Figure 5).

### Association between *Akt2* and *GLUT4* expression levels and iAUC at 12 weeks of age

There was a negative correlation between the iAUC of ITT results and *Akt2* mRNA expression (*r* = −0.61, *p* < 0.01) in the plantar muscle at 12 weeks of age. Although not significant, there was a tendency toward a negative correlation between the iAUC of ITT results and *GLUT4* mRNA (*r* = −0.39, *p* = 0.07), Akt2 protein (*r* = −0.39, *p* = 0.07), and GLUT4 protein (*r* = −0.41, *p* = 0.06) expression levels in the plantar muscle at 12 weeks of age (Table 1).

**Table 1:** Association between Akt2 and GLUT4 expression and the incremental area under the curve (iAUC) in the plantar muscle at 12 weeks of age.

			mRNA expression		Protein expression	
			*Akt2*	*Glut4*	Akt2	GLUT4
iAUC	OGTT	r	-0.33	-0.12	-0.36	-0.17
		p	0.13	0.58	0.10	0.45
	ITT	r	-0.61	-0.39	-0.39	-0.41
		p	< 0.01	0.07	0.07	0.06

### DNA methylation detection

**Figure 6:**
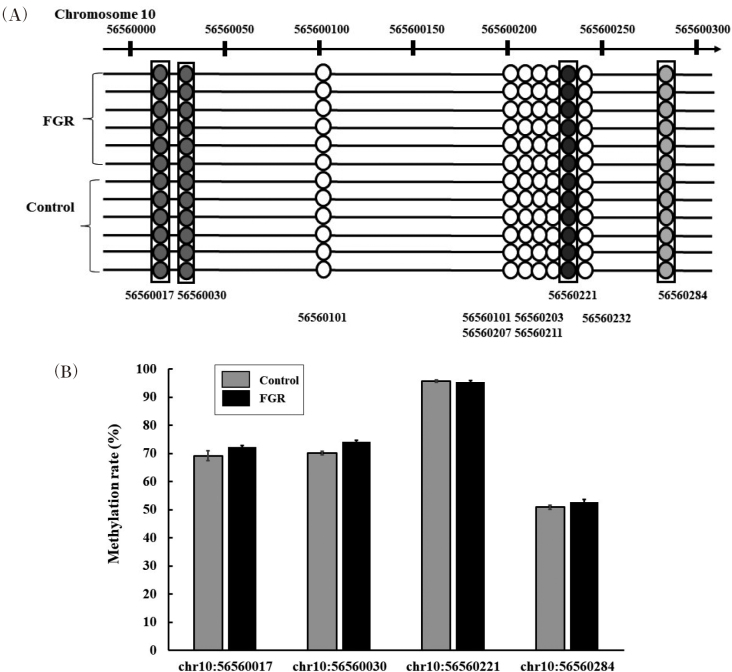
DNA methylation profiles in the promoter region of CpG sites of *GLUT4* in fetal growth restriction (FGR) and control rats (A). The methylation rate of *GLUT4* was not significantly different between the groups (B).

We examined the DNA methylation rate of four CpG sites (56560017, 56560030, 56560221, and 56560284) in *GLUT4* between the FGR and control groups (Figure 6A). There were no significant differences in the methylation rate between the groups (Figure 6B).

## Discussion

In this study, we investigated skeletal muscle insulin resistance in a rat model of maternal protein restriction during pregnancy. T2DM has been attributed to lifestyle and genetics; however, recent studies have indicated that a poor fetal environment is often associated with the development of glucose intolerance and insulin resistance later in life^19)^. Furthermore, excessive catch-up and obesity in FGR are associated with insulin resistance^20, 21)^. In this study, rats in the FGR group weighed less at birth than the controls; however, at 12 weeks of age, there was no significant difference between the groups. Moreover, the iAUCs of both blood glucose and insulin levels were significantly higher in the FGR group than in the control group at 12 weeks of age. Moreover, in the ITT, the iAUC of blood glucose level at 12 weeks of age was significantly higher in the FGR group than in the control group. Thus, protein-restricted FGR rats showed impaired blood glucose level-lowering ability in young adults without obesity.

In this study, we aimed to elucidate the mechanism of insulin resistance by analyzing the soleus and plantar muscles separately. Mammalian skeletal muscles are heterogeneous tissues composed of different fiber types identified by the expression of specific myosin heavy chain (MHC) isoforms. Muscle fibers can be classified into three distinct categories, namely, types I (slow twitch, oxidative), IIa (fast twitch, oxidative, glycolytic), and IIb (fast twitch, glycolytic). The action of insulin on glucose uptake and metabolism occurs in a muscle fiber- specific manner, with a greater response of insulin- stimulated glucose uptake observed in type I fibers than in type IIa or IIb fibers^22)^. The soleus muscle is mainly composed of type I fibers, plantar muscle is composed of type IIb fibers, and gastrocnemius muscle has different fibers in different areas^23, 24)^. In skeletal muscles, the decrease in protein synthesis due to fasting is greater in type IIb fibers than in type I and IIA fibers^25)^. Thus, in our study on a maternal protein-restricted diet model, the differences in the mRNA and protein levels of insulin signaling molecules can be seen more in the plantar muscle than in the soleus muscle and gastrocnemius muscle. Insulin-stimulated glucose transport is greater in skeletal muscle enriched in type I fibers^26)^, and this could be related to the higher GLUT4 level^27, 28)^. To the best of our knowledge, this study represents the first report to analyze the insulin-signaling molecules in the soleus and plantar muscles separately. Many other studies have analyzed the molecules in lower limb skeletal muscles of rat models without separating the muscles^17, 18, 29)^.

Reduced GLUT4 expression in skeletal muscles has been repeatedly observed in different experimental models of diabetes^30-33)^, similar to that in humans with insulin resistance and T2DM^34^^-^^36)^. Insulin resistance in the muscle tissue is associated with reduced levels of GLUT4^37)^. Some studies using FGR rats with maternal malnutrition have reported a decrease in GLUT4 levels in the skeletal muscles^11, 37)^. One study using a pig model also reported that offspring born to nutrient-restricted mothers showed reduced GLUT4 expression^38)^. Our results suggest that the reduced expression of GLUT4 in the plantar muscle may play an important role in skeletal muscle insulin resistance in young adults.

In rats, insulin signaling via Akt is reduced in offspring of dams exposed to a hypoxic or malnourished environment during pregnancy^39)^. Akt2 has been identified as the Akt isoform that is crucial for insulin-stimulated glucose uptake^40, 41)^. Xing et al.^29)^ reported that the reduction in GLUT4 expression is possibly mediated by decreased PI3K and phosphorylated Akt levels in maternal protein-restricted FGR models.

Several studies have reported DNA methylation due to nutritional abnormalities^25)^. In this study, the mRNA levels of insulin signaling pathway molecules in the skeletal muscles were significantly lower in the FGR group than in the control group. However, *GLUT4* methylation was not significantly different between the groups. In another study, the insulin-like growth factor 2 gene was differentially methylated in regions upstream of the entire gene and was found to modify downstream gene transcription^42)^. Another study reported that histone code modifications repress skeletal muscle GLUT4 transcription in the postnatal period, and that these changes persist in adult female FGR offspring^43)^. Thus, we speculate that the reduced mRNA expression of *GLUT4* in the skeletal muscle of FGR rats may be related to causes other than methylation^17)^.

Our study has some limitations. We could not show the causal relationship between insulin resistance and altered insulin signaling in skeletal muscles in our model. Although an evaluation of the activation/phosphorylation levels of Akt and PI3K might shed further light on the potential mechanism underlying insulin resistance, we could not evaluate the activation/phosphorylation levels of these proteins in this study; we attempted these experiments, but the results were not informative. Furthermore, we could not examine the alteration of skeletal muscle fiber types in this model. We could not analyze DNA methylation of *Akt2* and *PI3K* in the skeletal muscles. It was not possible to observe the influence of insulin resistance after 12 weeks of age.

In conclusion, protein-restricted FGR model rats showed insulin resistance in the skeletal muscles at 12 weeks of age without obesity. This indicates that abnormal insulin signaling in the skeletal muscles may cause insulin resistance. However, we were unable to demonstrate the involvement of DNA methylation in this model.

## Funding

This work was partially supported by KAKENHI (Grants-in-Aid for Scientific Research from the Japanese Ministry of Education, Culture, Sports Science and Technology; 21K07806) and by Subsidies for Current Expenditures to Private Institutions of Higher Education from the Promotion and Mutual Aid Corporation for Private Schools of Japan.

## Author contributions

Research conception and design: KA and HS; experiments: KA, YA, SI, and KT; statistical analysis of the data: YM; interpretation of the data: SI and TS; writing of the manuscript: KA and HS

All authors read and approved the final manuscript.

## Conflicts of interest statement

The authors declare that there are no conflicts of interest.
